# Sun-macerated *Hypericum perforatum* oleates in fixed oils: analytical characterization and comparative wound-healing activity

**DOI:** 10.55730/1300-0144.6190

**Published:** 2026-02-04

**Authors:** Cihan ÇAPAR, Esra KÜPELİ, Aylin YABA, Ayhan PARMAKSIZ, Engin SÜMER, Erdem YEŞİLADA

**Affiliations:** 1 Department of Pharmacognosy, Faculty of Pharmacy, Yeditepe University, İstanbul, Turkiye; 2 Department of Pharmacognosy, Faculty of Pharmacy, Gazi University, Ankara, Turkiye; 3 Department of Histology and Embryology, Yeditepe University School of Medicine, İstanbul, Turkiye; 4 Faculty of Medicine, Istanbul Health and Technology University, İstanbul, Turkiye; 5 Department of Pharmaceutical Toxicology, Faculty of Medicine, Yeditepe University, İstanbul, Turkiye

**Keywords:** *Hypericum perforatum*, fixed oils, oleates, fibroblast migration, wound healing

## Abstract

**Background/aim:**

*Hypericum perforatum* L. oleates prepared in fixed oils have long been used for wound management due to their antiinflammatory, antibacterial, and tissue-regenerative properties. The bioactivity of these oleates is mainly attributed to hypericin, hyperforin, and pseudohypericin. In the present study, the wound-healing potential of *H. perforatum* oleates prepared in different fixed oils, including *Olea europaea* L. (olive), *Sesamum indicum* L. (sesame), *Helianthus annuus* L. (sunflower), and *Nigella sativa* L. (black seed), is compared.

**Materials and methods:**

Fixed oils were characterized by gas chromatography–mass spectrometry, while oleuropein (olive oil) and thymoquinone (black seed oil) were quantified by high-performance liquid chromatography, and sesamin and sesamol (sesame oil) by spectrophotometry. Oleates were prepared using the traditional sun maceration method. Hypericin, hyperforin, and pseudohypericin were quantified by liquid chromatography–mass spectrometry and ultraviolet spectrophotometry. In vitro and in vivo assays were performed to assess the wound-healing activities of both the fixed oils and their respective oleates.

**Results:**

All oleates exhibited significantly greater wound-healing activity than the corresponding fixed oils. The most pronounced synergistic effects were observed with the olive oil and sesame oil oleates.

**Conclusion:**

Maceration of *H. perforatum* in fixed oils enhances the intrinsic therapeutic properties of oils and contributes to improvements in multiple wound-healing parameters. The findings of the present study support the traditional use of *H. perforatum* oleates and provide a scientific basis for their pharmacological development.

## 1. Introduction

Wounds are among the most common health concerns encountered in daily life, and different medicinal remedies have been developed and applied for their treatment since prehistoric times. Among these, *Hypericum perforatum* oleates prepared in different fixed oils have gained notable popularity. Traditionally, the fresh-flowering aerial parts of the plant are macerated in fixed oils for a defined period and stored as a household remedy for skin injuries, burns, or, when taken orally, for gastroesophageal ailments. Although olive oil is the most frequently used vehicle, other fixed oils—including sesame, black seed, and sunflower oils—are also employed. Preparation methods and maceration times can vary; however, the sun-maceration technique is the most commonly reported approach, typically lasting for 15–45 days [1].

The olive oil (OO) macerate of *H. perforatum* (HPO) contains such phloroglucinol derivatives as hyperforin, adhyperforin, and furohyperforin. Additionally, protohypericin and pseudoprotohypericin, belonging to the naphthodianthrone group, are believed to be converted into the more stable compounds hypericin and pseudohypericin during maceration [2]. Several pharmacological studies to date have confirmed the antiinflammatory and antimicrobial activities of these compounds. While the pharmacological potential of *H. perforatum* oleates prepared in other fixed oils—namely sesame (*Sesamum indicum* L.), black seed (*Nigella sativa* L.), and sunflower (*Helianthus annuus* L.)—has been experimentally investigated, there has been no comparative study to date systematically examining their analytical composition and wound-healing efficacy in parallel.

Medicinal plants and their bioactive constituents continue to be an important source of therapeutic agents, particularly for the management of inflammatory and tissue-damaging conditions. Recent pharmacological studies have highlighted the role of plant-derived secondary metabolites such as polyphenols, terpenoids, and phenolic acids in modulating inflammation, oxidative stress, and tissue regeneration pathways. Both traditional knowledge and contemporary experimental research support the relevance of phytochemically complex preparations in wound healing and inflammatory disorders, underscoring the need to investigate multicomponent botanical systems rather than isolated compounds alone [3].

We present here a comparative investigation of the chemical composition and wound-healing effects of selected fixed oils and their corresponding oleates, both in vitro and in vivo. The central hypothesis of this study is that the wound-healing activity of *H. perforatum* oleates is not attributable solely to the intrinsic effects of the plant constituents or the vehicle oils alone, but rather to a synergistic interaction between *H. perforatum*-derived compounds and the fixed-oil matrices. Accordingly, the objectives of the present study are (i) to compare the wound-healing efficacy of *H. perforatum* macerates with their corresponding vehicle oils, (ii) to evaluate the differential contributions of distinct vehicle oils, and (iii) to determine whether maceration enhances biological activity beyond the additive effects of their plant and oil components. To this end, fresh flowering aerial parts of *H. perforatum* were macerated in glass jars containing four fixed oils (olive, sesame, black seed, and sunflower) using the traditional sun-maceration method for 40 days. Hypericin, hyperforin, and pseudohypericin content were quantified by liquid chromatography–mass spectrometry (LC–MS) and ultraviolet (UV) spectroscopy, and fixed oil quantities by gas chromatography–mass spectrometry (GC–MS). Specific marker components—oleuropein in olive oil and thymoquinone in black seed oil—were determined by high-performance liquid chromatography (HPLC), while the sesamin and sesamol content of sesame oil were measured spectrophotometrically. In vitro and in vivo experiments were conducted to evaluate the wound-healing activities of both the fixed oils and their oleates. Following the in vivo wound-healing assays, histopathological evaluations of tissue samples were performed, and the effects of the test samples on extracellular matrix (ECM)-related enzymes were examined.

## 2. Materials and methods

### 2.1. Materials

*Hypericum perforatum* was collected from a cultivation area in Balıkesir, Türkiye, in June 2021. The botanical identification of the samples was confirmed by Dr. Erdinç Oğur (Ege Agricultural Research Institute, Department of Medicinal Plants). Voucher specimens were deposited in the Herbarium of the Ege Agricultural Research Institute, İzmir, Türkiye (ETAEIZ280621, sample no: 01,01). The plant materials were shade-dried at room temperature, protected from light, and stored in appropriate cabinets.

The vehicle fixed oils used in the study—olive oil (OO), black seed oil (NSO), sesame oil (SO), and sunflower oil (HAO)—were obtained from Nativital Doğal Sağlık Ürünleri, Istanbul, Türkiye (CAS numbers: 8001-25-0, 90064-32-7, 8008-74-0, 8001-21-6).

### 2.2. Preparation of oleates

Oleates of *H. perforatum* were prepared in four different fixed oils using the traditional sun-maceration method. Briefly, 125 g of freshly flowering aerial parts of *H. perforatum* were macerated in 500 g of the four fixed oils (OO, SO, NSO, and HAO). Traditionally, macerates should be exposed to sunlight during the day and stored under cooler conditions at night. To duplicate this process, the samples were placed under direct sunlight for 40 days and moved to a cool environment overnight, ensuring that the jars did not cast shadows on each other. After maceration, the mixtures were filtered to remove solid residues, yielding the final oleates. The extracts were stored in tightly sealed amber glass bottles, protected from light, and kept at room temperature.

### 2.3. Analysis of oleates and fixed oils

#### 2.3.1. Quantitative analysis of fixed oils

Prior to analysis, the acceptance criteria (specifications) for the base oils (OO, NSO, SO, and HAO) were established according to the European Pharmacopoeia and related standardization guidelines.

The fatty acid composition of the fixed oils was determined by GC–MS. The oil samples were derivatized with boron trifluoride (BF_3_) using the transmethylation method to produce fatty acid methyl esters (FAMEs). One microliter of each sample was injected (split ratio 40:1) into the GC–MS system (Agilent 7890B GC coupled with 5977B MSD). Separation was performed on an Agilent HP-Innowax FSC column (60 m × 0.25 mm i.d., 0.25 μm film thickness), with helium as the carrier gas (0.7 mL/min). Oven conditions were: initial 60 °C (10 min hold), ramped first to 220 °C at 4 °C/min (10 min hold), and then to 240 °C at 1 °C/min. The injector temperature was 250 °C. Mass spectra were recorded at 70 eV, with a scan range of 35–450 m/z. Compounds were identified by comparison with the Wiley GC–MS and TBAM Essential Oil Constituents libraries, and relative percentages were calculated from total ion chromatograms (TIC) [4].

A complementary GC–FID analysis was carried out on an Agilent 7890B GC system under the same chromatographic conditions to ensure identical elution order. The injector and detector (FID) temperatures were set to 250 °C.

##### 2.3.1.1. Determination of oleuropein content in OO

Five grams of the oil sample were transferred into a Falcon tube, and 15 mL of methanol was added. The mixture was vortexed at 2000 rpm for 2 min, followed by sonication in an ultrasonic bath for 1 min. The procedure was repeated, after which the sample was centrifuged at 5000 rpm for 5 min. The supernatant was collected, and the extraction procedure was repeated twice on the residue. All supernatants were combined and evaporated under reduced pressure. The residue was redissolved in 2.5 mL methanol and filtered through a 0.45 μm membrane filter prior to HPLC analysis [5].

Chromatographic separation was performed on an Agilent 1260 Infinity HPLC system equipped with an Inertsil ODS-4 column (5 μm, 4.6 × 250 mm). The mobile phase consisted of (A) 1% acetic acid/acetonitrile and (B) methanol (1:1, v/v), with isocratic elution (55% A, 45% B). The flow rate was 1 mL/min, and the total run time was 10 min. Detection was carried out at 280 nm, and oleuropein was identified at a retention time of 5.95 ± 0.1 min.

##### 2.3.1.2. Determination of lignan content in SO

Lignan content was determined according to the method proposed by Bhatnagar et al. [6]. Oil samples (0.01 g) were dissolved in 10 mL of a hexane–chloroform mixture (7:3, v/v), and absorbance was measured at 288 nm using a UV–Vis spectrophotometer (Libra). The solvent mixture served as the blank. Lignan content was calculated using the following formulas [6]:


$$\begin{array}{l} \%\;\text{Lignans }(\text{as sesamol})=\frac{A}{W}\times \frac{100}{230.1}\\ \%\;\text{Lignans }(\text{as sesamin})=\frac{A}{W}\times \frac{100}{231.1} \end{array}$$

where:

**A** = absorbance of the sample
**W** = weight of the sample (g/100 mL)
**230.1 and 231.1** = E1%1cm values for sesamol and sesamin, respectively.

##### 2.3.1.3 Determination of thymoquinone content in NSO

A 0.100 g test sample (±0.001 g) was weighed into a 10 mL volumetric flask, diluted to volume with hexane, and vortexed. Approximately 1 mL of the solution was transferred into a vial for HPLC analysis [7].

The analysis was carried out using an Agilent 1100 Series HPLC system equipped with a DAD detector and an Agilent Zorbax C18 column (5 μm, 4.6 × 150 mm). The mobile phase consisted of water:2-propanol:methanol (50:45:5, v/v/v), with a flow rate of 1 mL/min and an injection volume of 10 μL. Detection was performed at 254 nm under isocratic elution conditions.

### 2.4. Analysis of oleates

#### 2.4.1. General specifications of *H. perforatum* oleates and determination of hypericin content by UV spectroscopy

*H. perforatum* oleate (5 g) was mixed with refined corn oil (10 g). Absorbance was measured at 588 nm, using refined corn oil as the blank. Hypericin content was calculated using the following formula [8]:


$$\%\;\text{Hypericin}=\frac{ABS\times M_{1}\times 4.5}{M_{2}\times 1000}$$

where:

**ABS** = absorbance at 588 nm
**M_1_** = total weight of *H. perforatum* oil + corn oil (g)
**M_2_** = weight of *H. perforatum* oil alone (g)
**4.5** = spectroscopic factor

#### 2.4.2. Determination of hypericin, hyperforin, and pseudohypericin by LC–MS/MS

Oil samples (0.5 g) were extracted with 4.5 mL of chloroform:methanol (4:6, v/v). The extracts were filtered and analyzed using an LC–MS/MS system equipped with a Zorbax SB-C18 HT column (2.1 × 50 mm, 1.8 μm). The column temperature was maintained at 35 °C [9].

The mobile phase consisted of:

**(A):** water:methanol (95:5, v/v) containing 5 mM ammonium formate and 0.01% formic acid, and
**(B):** methanol containing 5 mM ammonium formate and 0.01% formic acid.

The flow rate was set at 0.150 mL/min. Pseudohypericin, hypericin, and hyperforin were quantified under optimized LC–MS/MS conditions (Table 1):

### 2.5. Bioactivity studies

#### 2.5.1. In vivo studies

##### 2.5.1.1. Animals

BALB/c mice were obtained from YÜDETAM. All procedures were carried out with the approval of the Yeditepe University Animal Experiments Local Ethics Committee (approval numbers: 2022-26 and 2022-008). The mice were acclimatized to laboratory conditions for 5 days prior to experimentation.

The number of animals used in this study was determined in accordance with ethical principles aimed at minimizing animal use while ensuring sufficient biological relevance. Group sizes were selected based on previously published wound-healing studies employing comparable excisional wound models and outcome measures. No formal a priori power analysis was conducted prior to the study.

##### 2.5.1.2. In vivo wound-healing activity model

The animals were randomly divided into three groups and housed individually. The scapular region was shaved one day prior to surgery and the body weights were recorded. Anesthesia was induced by intraperitoneal injection of xylazine (5 mg/kg) and ketamine (80 mg/kg). Following disinfection with povidone-iodine and alcohol, four full-thickness excisional wounds (5 mm diameter) were created bilaterally using a punch biopsy tool.

The treatment groups received topical applications of *H. perforatum* oleates prepared in olive oil (HPO), sesame oil (HP+SO), black seed oil (HP+NSO), sunflower oil (HP+HAO), or the respective vehicle oils alone (OO, SO, NSO, HAO). Isotonic saline was used as the negative control (NC), and Madecassol as the positive control (PC). All formulations were applied with a spatula to fully cover the wound surface. To minimize contamination, the cages were lined with fresh bedding.

Wound healing was documented by photographing the lesions on days 0, 4, 7, and 10. The wound areas were measured using ImageJ software. For analgesia, carprofen (3 mg/kg) was administered subcutaneously twice daily for 3 days. The animals were monitored daily, and weighed and euthanized on day 10 under high-dose anesthesia.

##### 2.5.1.3. Histopathological analysis

Excised tissue samples were fixed in 10% formalin for 6 h at room temperature, washed in tap water, and dehydrated in graded ethanol (70%, 80%, 90%, 100%). The samples were then cleared in xylene, embedded in paraffin, and sectioned at a thickness of 5 μm. The sections were stained with hematoxylin and eosin (H&E) and examined under a light microscope (Leica DM600).

The following parameters were evaluated: epidermal regeneration, epidermal thickness, granulation tissue formation, inflammatory cell infiltration, angiogenesis, fibroblast proliferation, collagen deposition, presence of hair follicles, and sebaceous gland activity. Semiquantitative scoring was applied as follows: (−/+) minimal; (+) slight; (++) moderate; (+++) extensive; and (−) absent.

##### 2.5.1.4. Statistical analysis

Quantitative data are presented as the mean ± standard deviation (SD). Time-dependent repeated measurements and group effects were analyzed using mixed-design analysis of variance (ANOVA). When a significant group × time interaction was observed, pairwise comparisons with negative and positive controls were conducted using the Bonferroni correction. In the absence of any interaction, the main time effects were interpreted. A p-value < 0.05 was considered statistically significant. The statistical analyses were performed using IBM SPSS Statistics, version 26.0 (IBM Corp., Armonk, NY, USA).

#### 2.5.2. In vitro studies

##### 2.5.2.1. Determination of hyaluronidase inhibitory activity

Hyaluronidase inhibition was evaluated by quantifying the release of N-acetylglucosamine from sodium hyaluronate, as described by Küpeli Akkol et al. (2022). Briefly, bovine hyaluronidase was dissolved in 0.1 M acetate buffer (pH 3.6) and incubated with varying concentrations of the test oils, previously dissolved in 5% dimethyl sulfoxide (DMSO). In the control group, 5% DMSO was used instead of the test samples. After incubation at 37 °C for 20 min, calcium chloride was added, and incubation continued for another 20 min. Sodium hyaluronate was then introduced and incubated for 40 min at 37 °C. The reaction mixture was subsequently treated with 0.4 M NaOH and 0.2 M sodium borate and incubated in a boiling water bath for 3 min. After cooling to room temperature, p-dimethylaminobenzaldehyde solution was added and incubated at 37 °C for 20 min to allow color development. Absorbance was recorded at 585 nm using a Beckmann Dual Spectrometer (Beckman, Fullerton, CA, USA) [10].

##### 2.5.2.2. Determination of collagenase inhibitory activity

Collagenase inhibition was assessed using *Clostridium histolyticum* collagenase (ChC). The test samples were dissolved in DMSO and mixed with enzyme solution in 50 mM Tricine buffer. After preincubation at 25 °C for 5 min, 2 mM N-[3-(2-furyl)acryloyl]-Leu-Gly-Pro-Ala (FALGPA) was added as the substrate. Each well, containing buffer, test sample, and enzyme, was incubated for 15 min. Decreases in optical density (OD) were measured at 340 nm using a spectrophotometer [10].

##### 2.5.2.3. Determination of elastase inhibitory activity

Elastase inhibition was determined using human neutrophil elastase (HNE). The test samples and enzyme were dissolved in 0.1 M Tris-HCl buffer (pH 7.5) and incubated at 25 °C for 5 min. N-Methoxysuccinyl-Ala-Ala-Pro-Val-p-nitroanilide (MAAPVN) was then added and incubated at 37 °C for 1 h, after which, the reaction was stopped with soybean trypsin inhibitor (1 mg/mL). P-nitroaniline release was measured at 405 nm. Enzyme inhibition (%) was calculated using the following formula:


$$\text{Inhibition }(\%)=\frac{OD_{\text{control}}-OD_{\text{sample}}}{OD_{\text{control}}}\times 100$$

where *OD*control and *OD*sample represent the absorbance values in the absence and presence of test samples, respectively [11].

## 3. Results and discussion

The in vivo and in vitro wound-healing effects of *H. perforatum* oleates prepared in olive (OO), sesame (SO), black seed (NSO), and sunflower (HAO) oils, together with their respective vehicle oils, were compared. The physicochemical properties, fatty acid composition, microbiological quality, and key bioactive constituents of the fixed oils used in the oleate preparation are summarized in Tables 2–4. All oils complied with the relevant pharmacopeial and international standards, confirming their suitability for pharmaceutical use. LC–MS and UV analyses confirmed the presence of hypericin, pseudohypericin, and hyperforin in all macerates. Hypericin contents exceeded the German Commission E Monographs threshold of ≥0.005% [12], with values of 0.011% (HP+HAO), 0.018% (HP+NSO), 0.0063% (HP+SO), and 0.0062% (HPO). Quantitative differences in hypericin, pseudohypericin, and hyperforin levels were observed depending on the type of fixed oil used in the maceration process (Table 5). The vehicle oils complied with relevant pharmacopeial standards (Ph. Eur. for OO, Codex CXS 210-1999 for SO and HAO, GSO 670/2014 for NSO), and fatty acid/microbial analyses confirmed their suitability.

As there were significant differences between the baseline wound areas (day 0), closure was analyzed in terms of percentage change (Table 6). Mixed-design ANOVA revealed significant improvements over time across all sites (Table 7). For right-up (RU) wounds, main effects of time and group were significant (p < 0.001, p = 0.001), with consistent improvement from days 4 to 10. Right-down (RD) wounds showed significant interactions (p = 0.001): HP+SO (58.4 ± 20.0) and HPO (55.0 ± 41.6) outperformed the negative controls (NC) (−8.9 ± 36.8) on day 4, while by day 10 most macerates achieved nearly complete closure, surpassing both NC and PC. Left-up (LU) wounds exhibited significant time and group effects, reaching 93.0 ± 14.2% by day 10. Left-down (LD) wounds also showed a significant interaction (p = 0.004) with HP+HAO, HP+SO, and HPO superior to NC on days 4 and 7, and all groups outperforming NC by day 10.

**Figure 1:**
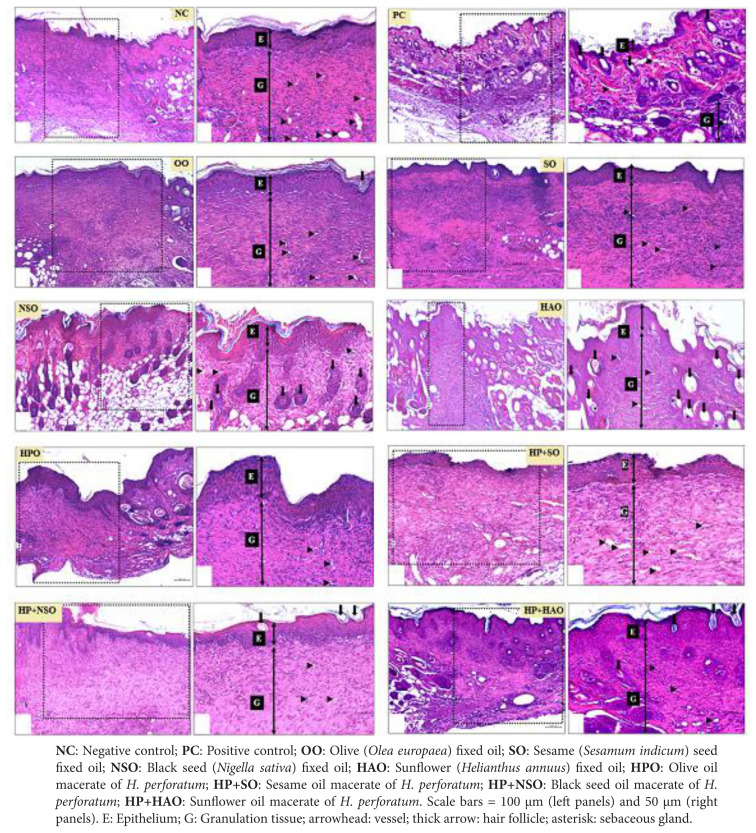
Representative histological sections of wound healing in mouse skin at postinjury day 10, with hematoxylin and eosin (H&E) staining.

Semiquantitative histological scoring (Table 8) and representative micrographs (Figure 1) revealed distinct profiles. NC wounds showed thick neo-epidermis, abundant inflammatory cells, and absent appendages, whereas PC achieved near-complete reepithelialization with normal epidermis, follicles, and glands. Among the vehicles, OO and SO supported near-normal epidermis with increased fibroblasts and collagen; NSO favored angiogenesis and follicle formation, while HAO yielded moderate repair with sparse appendages. Among the macerates, HPO induced strong fibroblast proliferation, collagen deposition, and controlled angiogenesis. HP+SO further increased capillary density and fibroblast activity, accompanied by follicle development. HP+NSO balanced moderate fibroblast/collagen stimulation with normal angiogenesis and antiinflammatory activity, and HP+HAO enhanced epidermal thickness, collagen, and fibroblasts, with observable appendages.

**Figure 2:**
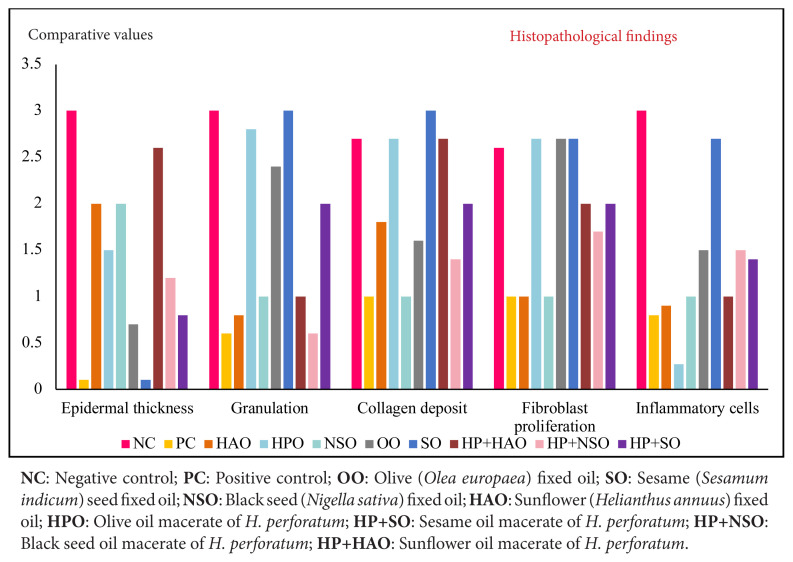
Comparison of histopathological findings of *H. perforatum* oleates and corresponding fixed oils in mouse skin at postinjury day 10.

Epidermal thickness was greatest in NC on day 10, with ongoing granulation suggesting hyper granulation and potential hypertrophic scarring (Figure 2). PC had the lowest epidermal thickness with minimal granulation and low fibroblast proliferation—adequate for minor wounds, but suboptimal for chronic wounds where stronger barrier formation is required [13]. Compared with OO, greater granulation and collagen deposition were observed with HPO, and while there were fewer inflammatory cells than SO and NC, the values were somewhat higher than for the other oils—potentially beneficial for orderly remodeling [14–16]. SO promoted substantial fibroblast/collagen proliferation and granulation but minimal epidermal thickening, which may be favorable for acute wounds [17, 18]. HP+SO approximated the in vivo efficacy of HPO/SO and improved inflammatory control versus SO alone, producing epidermal thickening compatible with barrier reinforcement. NSO demonstrated strong antiinflammatory activity, consistent with the antimicrobial/antiinflammatory profile of thymoquinone [19–21]. Alone, NSO had modest effects on collagen and fibroblasts. In contrast, HP+NSO increased both, while also maintaining reduced epidermal thickening, suggesting that maceration potentiates matrix building without impairing inflammation control—an advantageous profile for infected or high-bioburden chronic wounds [22]. HAO modestly increased collagen but lagged in fibroblast proliferation and granulation, and inflammation was well suppressed, consistent with linoleic acid-mediated actions [23, 24]. HP+HAO markedly boosted collagen and fibroblast proliferation while maintaining an antiinflammatory tone, and increased epidermal thickness without proportional rises in granulation, suggesting a lower risk of hypertrophic scarring while reinforcing the barrier [25–27].

**Figure 3:**
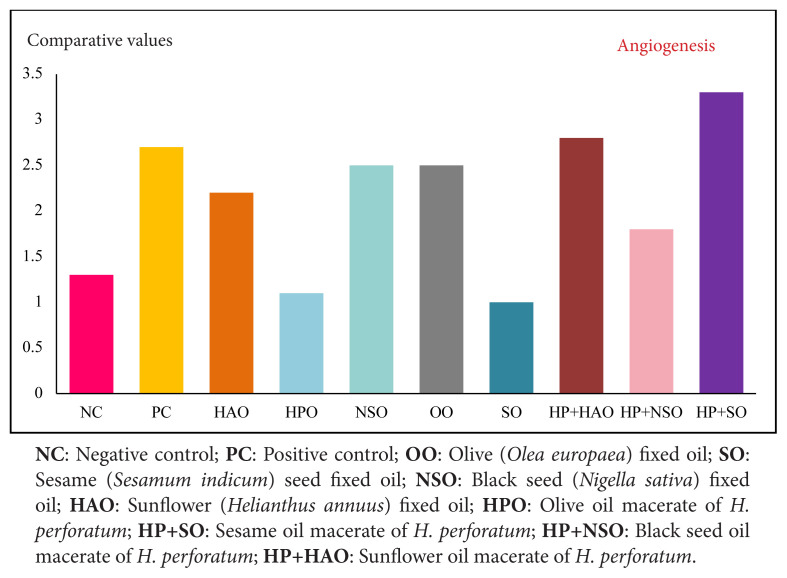
Comparison of angiogenesis in mouse skin wounds treated with *H. perforatum* oleates and corresponding fixed oils at postinjury day 10.

Angiogenesis was highest in HP+SO and lowest in HPO (Figure 3). Previous studies have reported antiangiogenic tendencies for HPO [28–33], which may benefit scar-minimizing healing or oncologic wounds where neovascularization is undesirable.

**Figure 4:**
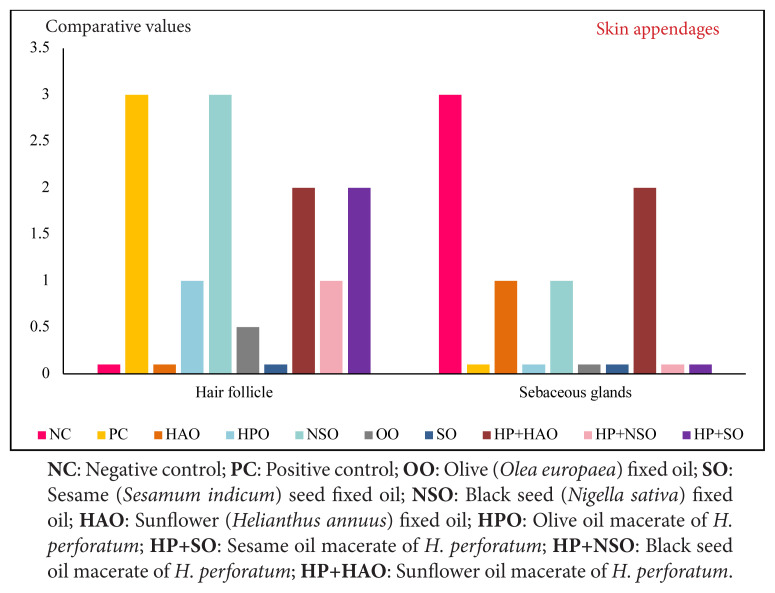
Histological comparison of hair follicle and sebaceous gland regeneration in oleates and fixed oils.

Hair follicle formation was most pronounced in PC and was also supported by NSO. HP+SO and HP+HAO promoted follicle development more strongly than their vehicle oils, paralleling their higher angiogenic activity. HPO induced fewer follicles but showed consistent epidermal repair, suggesting a balance between reepithelialization and appendage regeneration (Figure 4).

Oleates also achieved greater hyaluronidase and elastase inhibition than the vehicles, supporting balanced ECM turnover [34–38]. HPO and HP+SO showed the strongest hyaluronidase inhibition, while elastase inhibition was highest with HPO, HP+SO, and OO. Maceration increased elastase inhibition most in HAO (+49%) but reduced it in NSO compared with HP+NSO. Collagenase inhibition was moderate, with HPO ranking highest overall (Table 9).

Pseudohypericin, implicated as a key antiinflammatory constituent, was highest in HP+HAO and lowest in HPO; correspondingly, HP+HAO showed a high antiinflammatory response on day 10, whereas the response was lower with HPO. However, the overall pharmacology likely reflects multicomponent synergy rather than any single marker, as expected for botanical oleates [39–41]. OO contributes phenolics and squalene, which support angiogenesis and barrier repair [42–45]. SO offers polyunsaturated fatty acid (PUFA) and antioxidants that enhance moist wound healing [46, 47]. NSO, rich in thymoquinone, adds antimicrobial and antiinflammatory benefits [19–21, 48]. HAO, which is rich in linoleic acid, aids barrier restoration and modulates inflammation [49–51]. Importantly, hypericin, hyperforin, and pseudohypericin levels did not linearly predict bioactivity, underscoring the multicomponent synergy of botanical oleates [39]. Pseudohypericin, which was higher in HP+HAO, was correlated with strong antiinflammatory responses, while HPO’s restrained angiogenesis and epidermal thickening confirmed its suitability for scar-minimizing contexts.

Overall, HPO and HP+SO consistently achieved superior closure, favorable histology, and strong ECM enzyme modulation. HP+NSO enhanced infection control without impairing repair, while HP+HAO reinforced barrier formation with a lower risk of hypertrophic scarring.

From a clinical perspective, the present findings may inform the development of topical wound-care formulations based on *H. perforatum* oleates. The distinct biological profiles observed among different oleate preparations—such as differences in angiogenic response, inflammatory modulation, and barrier reinforcement—suggest that formulation-specific effects may influence wound-healing trajectories. An acute excisional wound model was used for the present study, and the findings provide a preclinical rationale for further evaluation of selected oleate formulations in disease-relevant experimental models and, ultimately, in clinical settings. Importantly, the observed enhancement of wound repair without evident adverse effects supports the potential translational value of these preparations as adjunctive or supportive topical therapies.

Despite these promising findings, it is important to acknowledge that the present study has certain limitations, one of which is the absence of an a priori statistical power analysis for animal group size determination. Although the number of animals was chosen in line with ethical guidelines and comparable experimental wound-healing studies, the lack of a formal power calculation may limit the ability of the study to detect subtle effect size differences between the treatment groups. Future studies incorporating prospective power analyses and larger cohorts would further strengthen statistical robustness.

In addition, although quantitative LC–MS/MS data for hypericin, hyperforin, and pseudohypericin were obtained, no formal statistical correlation analysis between individual marker concentrations and specific biological endpoints (such as angiogenesis, inflammatory cell infiltration, or epidermal thickness) was performed. This was due to the limited number of independent chemical data points per formulation and the semiquantitative nature of several histopathological parameters, which precluded a statistically robust correlation analysis. Consequently, the biological effects observed in this study are interpreted as resulting from multicomponent interactions rather than linear relationships with single marker compounds. Future studies employing larger sample sizes and fully quantitative biological readouts will be necessary to enable meaningful chemometric correlation analyses.

Considering the safety of the formulations, the fixed oils used in the present study (olive, sesame, black seed, and sunflower oils) are widely employed in topical and traditional medicines, and are generally regarded as safe when applied to intact or wounded skin. Similarly, *H. perforatum* oleates have a long history of topical use in folk medicine for the management of minor wounds and burns. In the present study, no adverse local reactions, signs of irritation, or abnormal behaviors were observed in the treated animals throughout the experimental period. While detailed toxicological evaluations and long-term safety assessments were beyond the scope of this study, they should be included in future investigations.

## 4. Conclusions

*Hypericum perforatum* oleates demonstrated distinct wound-healing profiles depending on the vehicle oil. HPO consistently supported fibroblast migration, collagen deposition, and controlled epidermal thickening, favoring scar-minimizing repair. HP+SO was associated with enhanced angiogenic responses and accelerated wound closure, while HP+NSO showed a balanced profile combining antiinflammatory effects with matrix support. HP+HAO primarily promoted barrier reinforcement with limited epidermal hyperplasia [13–18, 22–27].

Although these findings suggest that different oleate formulations may be suited to specific wound-healing contexts, such interpretations should be regarded as exploratory. As the present study employed an acute excisional wound model, extrapolation to complex clinical wound types (such as diabetic, ischemic, or infected chronic wounds) requires further validation in disease-specific experimental models and clinical studies [31–33, 52–56].

## Figures and Tables

**Table 1:** Gradient elution program and optimized LC–MS/MS conditions for the quantification of pseudohypericin, hypericin, and hyperforin.

Duration (minutes)	A (%)	B (%)
0.00	95	5
2.00	80	20
10.00	50	50
20.00	10	90
41.00	50	50
42.00	80	20

**Table 2:** Physicochemical properties and key bioactive compounds of fixed oils.

Parameter	Olive oil	Black seed oil	Sunflower oil	Sesame oil
**Appearance**	Conforms	Conforms	Conforms	Conforms
**Odor**	Conforms	Conforms	Conforms	Conforms
**Specific gravity (g/mL)**	0.913	0.920	0.921	0.920
**Refractive index**	–	1.469	1.465	1.467
**Acid value (mgKOH/g)**	0.10	5.20	0.60	0.50
**Peroxide value (meq O** **_2_** **/kg)**	0.30	14.20	1.50	1.20
**Unsaponifiable matter**	0.2%	–	3 g/kg	4 g/kg
**Iodine value**	–	–	82	113
**Oleuropein (μg/mL)**	155.44 ± 10.06	–	–	–
**Thymoquinone (μg/mL)**	–	3.84 ± 0.16	–	–
**Sesamin (%)**	–	–	–	1.23
**Sesamol (%)**	–	–	–	1.22

**Table 3:** Fatty acid composition (%) of fixed oils determined by GC–MS.

Fatty acid	Olive oil	Black seed oil	Sunflower oil	Sesame oil
**Palmitic acid (C16:0)**	12.6	12.5	6.1	9.8
**Stearic acid (C18:0)**	2.9	3.3	3.3	5.3
**Oleic acid (C18:1 n-9)**	67.5	23.0	33.2	39.0
**Linoleic acid (C18:2 n-6)**	11.6	55.6	55.0	43.5

**Table 4:** Microbiological quality assessment of fixed oils.

Test	Specification	Standard	Result
**Aerobic mesophilic count**	≤1000 cfu/g	Eur.Ph. 2.6.12	Conforms
**Yeast & mold**	≤100 cfu/g	Eur.Ph. 2.6.12	Conforms
***E. coli***	Negative	Eur.Ph. 2.6.13	Conforms
***Salmonella* spp**.	Negative	Eur.Ph. 2.6.13	Conforms

**Table 5:** Quantification of major bioactive compounds in *H. perforatum* oleates.

Macerate	UV	LC–MS/MS		
	Hypericin (%)	Hypericin (μg/g)	Pseudohypericin (μg/g)	Hyperforin (μg/g)
**HPO**	0.0062	0.160	0.050	17.520
**HP + HAO**	0.011	0.436	0.150	11.540
**HP + NSO**	0.018	0.687	0.120	33.460
**HP + SO**	0.0063	0.338	0.080	17.460

**HPO:** *H. perforatum* macerate in olive (*Olea europaea*) oil; **HP+HAO:** *H. perforatum* macerate in sunflower (*Helianthus annuus*) oil; **HP+NSO:** *H. perforatum* macerate in black seed (*Nigella sativa*) oil; **HP+SO:** *H. perforatum* macerate in sesame (*Sesamum indicum*) oil.

**Table 6:** Baseline (day 1) surface measurements of four wound sites across groups.

Groups	RU Day 1	RD Day 1	LU Day 1	LD Day 1
**NC**	6.91±2.35	7.99±1.65	6.56±2.35	6.81±4.10
**PC**	9.61±5.84	10.64±3.66	13.87±4.05	13.71±4.38
**HAO**	10.68±2.29	9.04±3.82	7.99±3.24	7.78±4.39
**HPO**	12.15±4.01	13.74±4.15	15.95±4.24	13.02±3.31
**NSO**	19.98±4.24	17.92±2.82	14.41±4.57	28.8±24.24
**OO**	17.79±5.03	9.37±4.51	15.99±7.97	22.03±4.71
**SO**	16.0±2.33	14.88±6.47	18.22±12.24	19.81±11.66
**HP+HAO**	10.42±2.73	12.78±3.56	11.34±4.94	10.38±4.54
**HP+NSO**	11.19±5.27	11.34±2.87	10.82±2.31	9.38±2.96
**HP+SO**	13.56±6.91	13.03±4.41	14.29±6.31	15.59±4.97
F	4.135	2.933	2.062	3.119
p	**<0.001**	**0.008**	**0.054**	**0.005**

**NC**: Negative control; **PC**: Positive control; **OO**: Olive (*Olea europaea*) fixed oil; **SO**: Sesame (*Sesamum indicum*) seed fixed oil; **NSO**: Black seed (*Nigella sativa*) fixed oil; **HAO**: Sunflower (*Helianthus annuus*) fixed oil; **HPO**: Olive oil macerate of *H. perforatum*; **HP+SO**: Sesame oil macerate of *H. perforatum*; **HP+NSO**: Black seed oil macerate of *H. perforatum*; **HP+HAO**: Sunflower oil macerate of *H. perforatum*. Values shown in **bold** indicate statistical significance (p < 0.05).

**Table 7:** Mixed-design ANOVA results for right-up (RU), right-down (RD), left-up (LU), and left-down (LD) wound site measurements.

*Right-up (RU)*						
Groups	Day 4	Day 7	Day 10	Group×time	Time	Group
				F (p)	F (p)	F (p)
**NC**	7.9±22.1	35.0±28.0	75.5±9.3	1.572 (0.127)	168.369 (<0.001)	4.045 (0.001)
**PC**	−4.6±65.1	83.6±18.2	91.3±9.8			
**HAO**	20.9±23.3	73.5±30.6	93.8±6.5			
**HPO**	51.8±33.7	99.5±1.2	100.0±0.0			
**OO**	−1.3±35.4	78.6±32.3	100.0±0.0			
**SO**	9.4±25.7	95.9±4.2	97.9±3.5			
**NSO**	16.1±11.8	66.9±15.6	95.9±9.2			
**HP+HAO**	23±14.8	76.2±9.5	98.2±4.4			
**HP+NSO**	27.8±36.4	90.6±9.2	100.0±0.0			
**HP+SO**	49.1±49.9	88.9±7.4	97.8±2.8			
**Total**	21.3±37.2^a^	79.6±24^b^	95.3±8.6^c^			
***Right-down (RD)***						
**NC**	−8.9±36.8	48.3±14.2	81.2±5.2	3.418 (0.001)	264.608 (<0.001)	6.116 (<0.001)
**PC**	44.2±18.3	72.2±7.6	83.6±9.4			
**HAO**	25.9±17.7	66.1±22.3	92.8±7.6			
**HPO**	55.0±41.6*	90.0±13.7*	100.0±0.0*,†			
**NSO**	10.9±12.0	55.1±19.6	99.3±1.5*,†			
**OO**	−9.2±26.4	76.6±17.7	92.8±9.9*			
**SO**	14.8±48.5	88.6±12.9*	100.0±0.0*,†			
**HP+HAO**	21.0±12.9	74.0±7.8	97.8±2.6*,†			
**HP+NSO**	41.1±17.3	91.9±9.6*	100.0±0.0*,†			
**HP+SO**	58.4±20.0*	90.2±10.9*	98.4±3.9*,†			
**Total**	26.7±34.3	76.1±19.5	94.9±8.1			
***Left-up (LU)***						
**NC**	−7.0±29.2	28.6±49.7	65.9±25.3	1.514 (0.131)	196.695 (<0.001)	4.954 (<0.001)
**PC**	8.7±24.0	66.0±17.0	91.9±8.8			
**HAO**	−7.1±55.6	63.6±25.8	96.6±5.7			
**HPO**	33.4±39.5	99.2±2.0	100.0±0.0			
**NSO**	−24.4±17.8	39.4±42.0	95.7±3.1			
**OO**	21.5±40.2	82.9±26.0	87.8±25.8			
**SO**	18.1±9.7	84.4±15.3	97.3±2.7			
**HP+HAO**	25.1±29.2	79.6±9.9	93.9±6.7			
**HP+NSO**	37.2±27.9	89.8±10.8	99.2±2.1			
**HP+SO**	36.7±11.1	82.2±23.1	98.1±2.2			
**Total**	15.6±34.8^a^	72.8±31.3^b^	93.0±14.2^c^			
***Left-down (LD)***						
**NC**	−20.0±34.6	−0.8±100.1	61.7±18.6	2.498 (0.004)	131.47 (<0.001)	6.041 (<0.001)
**PC**	36.4±39.6	63.9±15.6	88.6±8.7*			
**HAO**	3.7±18.2	55.5±38.8	88.4±18.1*			
**HPO**	56.1±13.3*	91.0±8.7*	99.5±1.2*			
**NSO**	−9.6±23.8	17.6±39.9	92.2±5.7*			
**OO**	25.6±20.9	63.6±28.8	90.9±7.0*			
**SO**	3.8±30.8	79.5±13.2	99.6±0.8*			
**HP+HAO**	42.8±24.2*	90.1±5.7*	98.8±1.9*			
**HP+NSO**	20.3±33.6	82.6±9.9*	93.1±8.7*			
**HP+SO**	62.0±25.2*	82.0±19.8*	96.9±3.8*			
**Total**	23.8±36.4	64.3±44.8	91.4±13.4			

**NC**: Negative control; **PC**: Positive control; **OO**: Olive (*Olea europaea*) fixed oil; **SO**: Sesame (*Sesamum indicum*) seed fixed oil; **NSO**: Black seed (*Nigella sativa*) fixed oil; **HAO**: Sunflower (*Helianthus annuus*) fixed oil; **HPO**: Olive oil macerate of *H. perforatum*; **HP+SO**: Sesame oil macerate of *H. perforatum*; **HP+NSO**: Black seed oil macerate of *H. perforatum*; **HP+HAO**: Sunflower oil macerate of *H. perforatum*.

* Statistically significant difference was found with the negative control group.

† Statistically significant difference was found with the positive control group.

**Table 8:** Semiquantitative scoring of histological parameters for wound-healing assessment.

Histological parameter	Experimental group									
	NC	PC	HAO	HPO	NSO	SO	OO	HP+HAO	HP+NSO	HP+SO
**Epidermal regeneration**	++	+++	+++	+++	+++	+++	+++	+++	+++	+++
**Epidermal thickness**	+++	−	++	++	++	−	−/+	++	+	+
**Granulation tissue**	+++	−/+	+	+++	+	+++	++	+	+	++
**Inflammatory cells**	+++	−/+	+	+++	+	+++	+	+	++	+
**Angiogenesis**	+	+++	++	+	++	+	++	+++	++	+++
**Proliferation of fibroblast**	+++	+	+	+++	+	+++	+++	++	++	++
**Collagen deposit**	+++	+	++	+++	+	+++	++	+++	+	++
**Hair follicle**	−	+++	+	+	+++	−	−/+	++	+	++
**Sebaceous glands**	−	+++	+	−	+	−	−	++	−	−

(−/+), almost none; (+), slight; (++), moderate; (+++), extensive; (−), absence. **NC**: Negative control; **PC**: Positive control; **OO**: Olive (*Olea europaea*) fixed oil; **SO**: Sesame (*Sesamum indicum*) seed fixed oil; **NSO**: Black seed (*Nigella sativa*) fixed oil; **HAO**: Sunflower (*Helianthus annuus*) fixed oil; **HPO**: Olive oil macerate of *H. perforatum*; **HP+SO**: Sesame oil macerate of *H. perforatum*; **HP+NSO**: Black seed oil macerate of *H. perforatum*; **HP+HAO**: Sunflower oil macerate of *H. perforatum*.

**Table 9:** Inhibitory activities of the test materials on hyaluronidase, collagenase, and elastase enzymes.

Material	Concentration (μg/mL)	Hyaluronidase inhibition (%) ± S.E.M.	Collagenase inhibition (%) ± S.E.M.	Elastase inhibition (%) ± S.E.M.
**HAO**	100	9.27±1.09	7.06±2.14	11.43±1.70
**HPO**	100	41.33±1.87**	16.13±0.81	31.29±0.93**
**NSO**	100	7.08 ±1.92	9.24±1.51	17.92±1.84
**SO**	100	14.11±1.27	7.31±1.62	22.08±1.64
**OO**	100	15.21±2.14	11.78±2.08	26.13±1.07*
**HP+HAO**	100	21.05±2.38	13.62±1.57	17.05±1.42
**HP+NSO**	100	19.25±1.63	15.26±2.13	13.24±1.16
**HP+SO**	100	29.16±1.57*	13.04±1.96	25.70±1.54*
**Tannic acid**	100	82.08±1.14***	-	-
**Epigallocatechin gallate**	100	-	38.09±0.74**	73.18±1.27***

* p < 0.05;

** p < 0.01;

*** p < 0.001;

S.E.M.: Standard error of the mean.

**OO**: Olive (*Olea europaea*) fixed oil; **SO**: Sesame (*Sesamum indicum*) seed fixed oil; **NSO**: Black seed (*Nigella sativa*) fixed oil; **HAO**: Sunflower (*Helianthus annuus*) fixed oil; **HPO**: Olive oil macerate of *H. perforatum*; **HP+SO**: Sesame oil macerate of *H. perforatum*; **HP+NSO**: Black seed oil macerate of *H. perforatum*; **HP+HAO**: Sunflower oil macerate of *H. perforatum*.
